# Deficiency of Retinaldehyde Dehydrogenase 1 Induces BMP2 and Increases Bone Mass *In Vivo*

**DOI:** 10.1371/journal.pone.0071307

**Published:** 2013-08-09

**Authors:** Shriram Nallamshetty, Hong Wang, Eun-Jung Rhee, Florian W. Kiefer, Jonathan D. Brown, Sutada Lotinun, Phuong Le, Roland Baron, Clifford J. Rosen, Jorge Plutzky

**Affiliations:** 1 Brigham and Women’s Hospital, Harvard Medical School, Boston, Massachusetts, United States of America; 2 Massachusetts General Hospital, Harvard Medical School, Boston, Massachusetts, United States of America; 3 Department of Oral Medicine, Infection, and Immunity, Harvard School of Dental Medicine, Boston, Massachusetts, United States of America; 4 Center for Clinical & Translational Research, Maine Medical Center Research Institute, Scarborough, Maine, United States of America; National Institutes of Health, United States of America

## Abstract

The effects of retinoids, the structural derivatives of vitamin A (retinol), on post-natal peak bone density acquisition and skeletal remodeling are complex and compartment specific. Emerging data indicates that retinoids, such as all trans retinoic acid (ATRA) and its precursor all trans retinaldehyde (Rald), exhibit distinct and divergent transcriptional effects in metabolism. Despite these observations, the role of enzymes that control retinoid metabolism in bone remains undefined. In this study, we examined the skeletal phenotype of mice deficient in retinaldehyde dehydrogenase 1 (Aldh1a1), the enzyme responsible for converting Rald to ATRA in adult animals. Bone densitometry and micro-computed tomography (µCT) demonstrated that Aldh1a1-deficient (*Aldh1a1^−/−^*) female mice had higher trabecular and cortical bone mass compared to age and sex-matched control C57Bl/6 wild type (WT) mice at multiple time points. Histomorphometry confirmed increased cortical bone thickness and demonstrated significantly higher bone marrow adiposity in *Aldh1a1^−/−^* mice. In serum assays, *Aldh1a1^−/−^* mice also had higher serum IGF-1 levels. *In vitro*, primary *Aldh1a1^−/−^* mesenchymal stem cells (MSCs) expressed significantly higher levels of bone morphogenetic protein 2 (BMP2) and demonstrated enhanced osteoblastogenesis and adipogenesis versus WT MSCs. BMP2 was also expressed at higher levels in the femurs and tibias of *Aldh1a1^−/−^* mice with accompanying induction of BMP2-regulated responses, including expression of Runx2 and alkaline phosphatase, and Smad phosphorylation. *In vitro*, Rald, which accumulates in *Aldh1a1^−/−^* mice, potently induced BMP2 in WT MSCs in a retinoic acid receptor (RAR)-dependent manner, suggesting that Rald is involved in the BMP2 increases seen in Aldh1a1 deficiency *in vivo*. Collectively, these data implicate Aldh1a1 as a novel determinant of cortical bone density and marrow adiposity in the skeleton *in vivo* through modulation of BMP signaling.

## Introduction

Retinoids - the products of retinol (vitamin A) and β carotene metabolism - direct fundamental cellular processes and play a crucial role in limb patterning and skeletal development [1]. Several lines of evidence indicate that retinoids also influence endochondral bone beyond development; however, the molecular basis of post-natal retinoid actions in bone remains poorly understood. Early pre-clinical studies in rodent models linked hypervitaminosis A with skeletal abnormalities and increased bone fragility [2], while human epidemiological studies identify high retinol intake as a risk factor for hip fractures in the elderly [3], [4]. Animal studies demonstrate that retinol and its main metabolite all trans retinoic acid (ATRA) consistently increase bone fragility in rodent models by reducing radial bone growth and bone density [5]–[8]. *In vitro* studies of retinoid regulation in bone cells have yielded more conflicting results. Retinoids have been reported to either induce or inhibit *in vitro* osteoblastogenesis and osteoclastogenesis depending on the differentiation marker examined and the cell system employed [9]–[16].

A recent study demonstrated that ATRA may also modulate fundamental cell fate decisions in the marrow niche. ATRA exerted divergent effects on osteoblastogenesis and adipogenesis in mesenchymal stem cells (MSCs), the common progenitor of marrow osteoblasts and adipocytes [17]. While ATRA induced the osteoblast marker alkaline phosphatase (ALP), it blocked adipogenesis in CH310T1/2 MSCs through a bone morphogenetic protein 2 (BMP2)-dependent pathway. Furthermore, the opposing effects of ATRA on osteoblastogenesis and adipogenesis in MSCs appear to depend on ATRA-mediated induction of Smad3 [18], [19], a downstream transcriptional mediator of BMP signaling pathways. BMP2, a member of the transforming growth factor (TGF)- β superfamily of cytokines, acts in a paracrine and autocrine manner to promote MSC osteoblastogenesis and enhance the osteogenic activity of differentiated osteoblasts [20], [21]. Interestingly, beyond its essential role in bone formation, BMP2 also promotes adipogenesis in CH310T1/2 mesenchymal cells [22]–[24].

Multiple structurally distinct retinoids exist that exert divergent biologic effects. As such, a complex system of metabolizing enzymes and transport proteins governs retinoid generation and free levels in a precise, controlled manner [25], [26]. Retinol is inactive and must be metabolized in order to exert its pleotropic actions. Retinol is first converted to all trans retinaldehyde (Rald) by alcohol dehydrogenases (ADHs). Subsequently, Rald is converted to ATRA, the main structural derivative of retinol, through the action of retinaldehyde dehydrogenase 1 (Aldh1) isoforms. While Aldh1a2 and Aldh1a3 control fundamental aspects of retinoid metabolism during embryonic development, Aldh1a1 is the main enzyme responsible for converting Rald to ATRA in adult animals and is the only isoform whose deficiency is not embryonically lethal [27].

Given Aldh1a1’s role as the rate-limiting enzyme in the biogenesis of ATRA *in vivo*, we hypothesized Aldh1a1 functions as a potentially novel determinant of fundamental aspects of skeletal remodeling and bone metabolism. To investigate this, we performed skeletal phenotyping of Aldh1a1-deficient (*Aldh1a1^−/−^*) mice and examined the effect of Aldh1a1 deficiency on MSC differentiation. We demonstrate here that female *Aldh1a1^−/−^* mice have higher cortical bone density and greater marrow adiposity when compared to age and sex matched wild type controls (WT). In exploring mechanisms underlying this coordinated change in bone density and marrow adiposity, we found primary *Aldh1a1^−/−^* MSCs express higher levels of BMP2 and demonstrate enhanced osteoblastogenesis and adipogenesis *in vitro*. In addition, *Aldh1a1^−/−^* mice express higher levels of BMP2 and its downstream targets such as runt-related transcription factor 2 (Runx2) and alkaline phosphatase (ALP) in their femurs and tibias compared to WT controls. In keeping with these effects, stimulation of WT MSCs with Rald, which accumulates *in vivo* in *Aldh1a1^−/−^* mice, induced BMP2 expression in a retinoic acid receptor (RAR)-dependent manner. Taken together, these data identify Aldh1a1 as a novel determinant of cortical bone formation through it's action on BMP2.

## Materials and Methods

### Mice

*Aldh1a1^−/−^* mice and C57BL/6 mice were housed at Harvard Medical School. The *Aldh1a1^−/−^* mice were originally generated on a C57BL/6 background and provided by Dr. Gregg Duester, Burnham Institute, La Jolla, CA [28]. Developmental phenotyping studies were carried out on age and sex-matched female wild type control (WT) and *Aldh1a1^−/−^* mice that were maintained at 25°C on a 12-hour light-dark cycle on standard chow diet (10.6% kcal fat PicoLab 20 Rodent diet 5506, Vitamin A content of 15 IU/g of diet).

### Ethics Statement

This study was carried out in strict accordance with the recommendations in the Guide for the Care and Use of Laboratory Animals of the National Institutes of Health. The protocol was approved by the Harvard Medical School Institutional Animal Care and Use Committee (Protocol 03998). Bones and tissues were harvested after euthanasia by Harvard IACUC-approved CO2 inhalation protocols and all efforts were made to minimize suffering.

### Dual-energy x-ray Absorptiometry (DXA)

Dual-energy x-ray absorptiometry (DXA) scanning was performed using the PIXImus system (GE-Lunar, Madison, WI) as previously described [29]. The PIXImus was used to assess femoral bone mineral density (BMD), and bone mineral content (BMC) in WT and *Aldh1a1^−/−^* mice at multiple developmental time points including 8 weeks (WT n = 5, *Aldh1a1^−/−^* n = 5), 12 weeks (WT n = 20, *Aldh1a1^−/−^* n = 18), 18 weeks (WT n = 10, *Aldh1a1^−/−^* n = 10), 26 weeks (WT n = 10, *Aldh1a1^−/−^* n = 9), and 36 weeks (WT n = 4, *Aldh1a1^−/−^* n = 4). A phantom standard provided by the manufacturer was assessed each day for instrument calibration.

### Micro-computed Tomography (µCT)

Microarchitecture of the trabecular bone and midshaft cortical bone of the femur was analyzed by µCT (resolution 10 µm, VivaCT-40; Scanco Medical AG, Bassersdorf, Switzerland) at multiple developmental time points including 6 weeks (WT n = 5, *Aldh1a1^−/−^* n = 5), 8 weeks (WT n = 5, *Aldh1a1^−/−^* n = 5), 12 weeks (WT n = 20, *Aldh1a1^−/−^* n = 18), 18 weeks (WT n = 10, *Aldh1a1^−/−^* n = 10), 26 weeks (WT n = 10, *Aldh1a1^−/−^* n = 9), and 36 weeks (WT n = 4, *Aldh1a1^−/−^* n = 4). Bones were scanned at an energy level of 55 kVp and intensity of 145 µA. Trabecular bone volume fraction and microarchitecture were evaluated starting approximately at 0.6 mm proximal to the distal femoral growth plate and extending proximally 1.5 mm. Measurements included bone volume/total volume (BV/TV), trabecular number (Tb.N.), trabecular thickness (Tb.Th.), and trabecular spacing. Scans for the cortical region were measured at the midpoint of each femur, with an isotropic pixel size of 21 µm and slice thickness of 21 µm, and used to calculate the average BA, total cross-sectional area, BA/total cross-sectional area, and Ct.Th. All scans were analyzed using manufacturer software (version 4.05; Scanco Medical AG).

### Histomorphometry

Twelve week-old female WT (n = 5) and *Aldh1a1^−/−^* (n = 5) mice were subcutaneously injected with 20 mg/kg calcein and 40 mg/kg demeclocycline on days 7 and 2 before necropsy, respectively. Tibias were removed and embedded without demineralization in methyl methacrylate. Undecalcified sections were cut at a thickness of 5 µm and mounted unstained for dynamic measurements (i.e., mineral apposition rate [MAR], mineralizing surface (MS/BS) and bone formation rate expressed per bone surface [BFR/BS], bone volume [BFR/BV], and tissue volume referent [BFR/TV]). Consecutive sections were stained with toluidine blue and TRAP to quantify osteoblast number and osteoclast number, respectively. Adipocyte number was counted using toluidine blue–stained sections. Histomorphometric analysis was performed using the OsteoMeasure system (Osteometrics Inc.), and the results were expressed according to standardized nomenclature [30].

### Bone Marrow Cell Isolation

Bone marrow stromal cells were isolated as previously described [31]. Briefly, tissue was dissected away from femurs and tibiae of age and sex matched WT and *Aldh1a1^−/−^* mice. Bone marrow was then flushed from the bones with αMEM media (Life Technologies) supplemented with 10% lot-selected Hyclone fetal bovine serum (FBS) (VWR International) and 1% penicillin-streptomycin (Life Technologies). Cells were then strained with 70 µM filter and then seeded for CFU-F, differentiation, and retinoid cellular assays as outlined below.

### Colony Forming Unit-Fibroblast (CFU-F) Assay

Bone marrow cells were isolated from WT and *Aldh1a1^−/−^* mice and plated at a density of 1×10^6^ cells/cm^2^. Cells were cultured in basal media consisting of αMEM media (Life Technologies) supplemented with 10% Hyclone fetal bovine serum (VWR International) and 1% penicillin-streptomycin (Life Technologies) for 7 days and then stained with crystal violet to enumerate CFU-F [32]. For quantitative CFU-F assays, primary WT and *Aldh1a1^−/−^* marrow cells were plated in 96 well dishes (10^5^ cells/well) and cultured in basal media for 14 days. Crystal violet staining was performed on day 14, and a well was considered positive for CFU-F if it contained greater than 20 crystal violet stained cells.

### Retinoid Treatment of MSCs

For the retinoid stimulation studies, WT primary marrow stromal cells were plated at a density of 1×10^6^ cell/cm^2^ in 12 well dishes and cultured in basal media for 10–14 days till confluence and then treated for 24 hours with the following: (1) DMSO (control); (2) Rald (Sigma) at concentrations of 100 nM, 500 nM, and 1 µM; (3) ATRA (Sigma) across a concentration range of 100 nM, 500 nM, and 1 µM; (4) Diethylaminobenzaldehyde (DEAB, Sigma) at a concentration of 1 µM; (5) Rald (1 µM) and DEAB (1 µM); (6) AGN 193109 (Sigma) at a concentration of 1 µM); (7) AGN (1 µM) and Rald (1 µM); (8) HX531 (a kind gift from Dr. Hiroyuki Kagechika, University of Tokyo, Japan) at a concentration of 1 µM; and (9) HX531 (1 µM) and Rald (1 µM). At the conclusion of the 24 hour treatment, RNA was isolated from the cells as described below (see “Reverse Transcriptase PCR and quantitative PCR” section below). All stimulations were performed in triplicate, and validated in a total of 3 independent experiments.

### *In vitro* MSC Osteoblastogenesis Differentiation

*In vitro* osteoblastogenesis assays were performed by plating marrow stromal cells isolated as described above at a density of 10×10^6^ cell/well in 6 well dishes (1×10^6^ cells/cm^2^). The cells were grown in basal media for 7 days, and then osteogenic media (basal media supplemented with ascorbic acid at 50 µg/mL and beta-glycerol phosphate at 10 mM) for another 7–14 days. Alkaline phosphatase (ALP) staining (Sigma 86R-KT) and measurement of ALP activity (Bioassay Systems, DALP-250) were performed after 7 days of culture in osteogenic media. After 14 days of culture in osteogenic media, alizarin red staining [33] and calcium content measurements (Bioassay Systems, DALP-250) were performed. Differentiation assays were performed in triplicate, and validated in a total of 3 independent experiments.

### *In vitro* MSC Adipogenesis Differentiation

Marrow cells were seeded in 24 well dishes (1×10^6^ cells/cm^2^) and treated with adipogenesis induction media (basal media supplemented with rosiglitazone 1 µM [Cayman Chemicals], insulin 10 µg/mL [Sigma Aldrich], dexamethasone 1 nM [Sigma Aldrich], and IBMX 0.5 mM [Sigma Aldrich]) on day 10 for 48 hours followed by adipogenesis maintenance media (basal media supplemented with rosiglitazone 1 µM, insulin 10 µg/mL) for 5 days. Oil Red O (ORO) staining was performed and quantified by counting ORO-positive cells in 5 random high power fields per genotype. Quantification of *in vitro* adipogenesis was also performed using the AdipoRed Assay (Lonza) according to the manufacturer’s protocol. Differentiation assays were performed in triplicate, and validated in a total of 3 independent experiments.

### MSC Proliferation

Marrow cells (2 × 10^4^ cells/well) were cultured in 96-well plates and proliferation was measured using BrdU incorporation (Roche Applied Science) at day 3,7, and 10 of culture according to the manufacturer's protocol.

### Reverse Transcriptase PCR (RT-PCR) and Quantitative Real Time PCR (qPCR)

RNA was harvested from cells using Trizol (**Life Technologies**). For gene expression analysis in whole bone, RNA was isolated from one femur and tibia from matched WT controls and *Aldh1a1^−/−^* female mice (n = 10–12 per genotype) using the Qiagen TissueLyzer II System according to the manufacturer’s protocol. RT-PCR was performed with 0.5–1 µg of RNA using the High Capacity cDNA synthesis kit (Applied Biosystems, Bedford, MA). Gene expression analysis was performed using an iQ quantitative real time thermal cycler system (Bio-Rad). One µL of diluted cDNA (diluted 1∶5) generated from RT-PCR was then used as template for qPCR amplification using 2X iQ SYBR green mastermix (Bio-Rad) in a total reaction volume of 25 µL. The delta-delta Ct method (DDCt) was employed in qPCR analysis to determine relative changes in mRNA expression. The ribosomal gene 36B4 was used as the internal control gene.

### Western Blot

Cell lysates were collected using RIPA buffer (Boston Bioproducts Inc). For whole bone samples, femur and tibias were dissected and immediately flash frozen in liquid nitrogen (n = 4 per genotype). The frozen bone samples were then crushed using a BioPulverizer (Biospec Products) and the fragments were then homogenized in RIPA buffer using the TissueLyzer system (Qiagen). Western blot analysis was carried out as described [34] using Aldh1a1 (AbCam), GAPDH (Santa Cruz Biotechnology), Phospho-Smad1–5–8, Smad 1, beta-actin (Cell Signaling Technologies) antibodies.

### Statistical Analysis

All data are presented as means ± standard deviations. Unpaired student t-tests were employed in the statistical analysis of WT and *Aldh1a1^−/−^* skeletal phenotyping data (PIXImus densitometry, µCT, histomorphometry), and *in vitro* assays (CFU-F Giemsa quantification, ALP activity, calcium measurements, Adipored assay for quantification of adipogenesis, and gene transcript expression analysis). For the retinoid treatments of WT marrow stromal cultures, a two way ANOVA statistical method was used in the analysis of differences between BMP2 mRNA expression in samples treated with DMSO versus retinoids and retinoid receptor modulators (see above). Post hoc analysis for significance of difference between the means of each treatment group was performed using the Tukey multiple comparisons test.

## Results

### Aldh1a1 is the Predominant Aldh isoform Expressed in Bone *in vivo*

**Figure 1:**
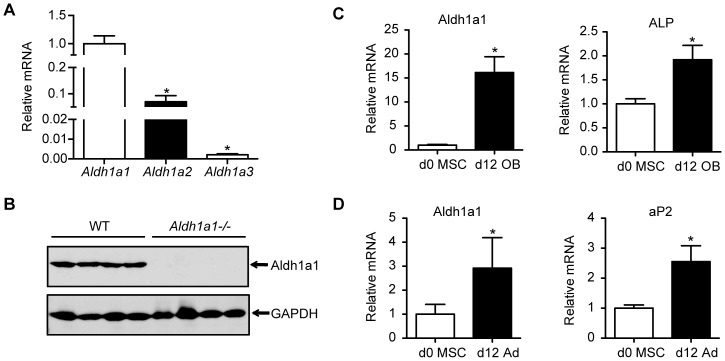
Aldh1a1 expression in bone. A. Quantitative real time PCR revealed Aldh1a1 as the predominant Aldh1 isoform expressed in the long bones (femur and tibia) of WT mice. B. Western blot analysis of whole bone from WT and *Aldh1a1^−/−^*mice (n = 4 per genotype). No Aldh1a1 was detectable in femurs and tibias of *Aldh1a1^−/−^*mice. C. Aldh1a1 expression during primary MSC osteoblastogenesis. Aldh1a1, along with the osteoblast marker alkaline phosphatase (ALP), are induced during *in vitro* MSC differentiation into osteoblast (OB) lineage. D. Aldh1a1 expression during primary MSC adipogenesis. Aldh1a1 and aP2, a marker of mature adipocytes, are induced during *in vitro* MSC differentiation into an adipocyte (Ad) lineage. *p<0.05.

Given the existence of three Aldh1 isoforms, we examined expression of Aldh1a1, 2 and 3 mRNA levels in whole bone. Aldh1a1 was the primary Aldh isoform expressed in the femurs and tibias of WT female mice (Figure 1A). As expected, Aldh1a1 protein was present in the long bones of WT but not *Aldh1a1^−/−^* mice (Figure 1B). We then examined Aldh1a1 expression patterns during *in vitro* MSC osteoblastogenesis and adipogenesis. Aldh1a1 is expressed at low levels in primary WT MSCs derived from 12 week old mice but is induced during differentiation of MSCs into osteoblast (Figure 1C) and adipocyte lineages *in vitro* (Figure 1D).

### Aldh1a1 Deficiency Increases Bone Mineral Density at Multiple Developmental Time Points

**Figure 2:**
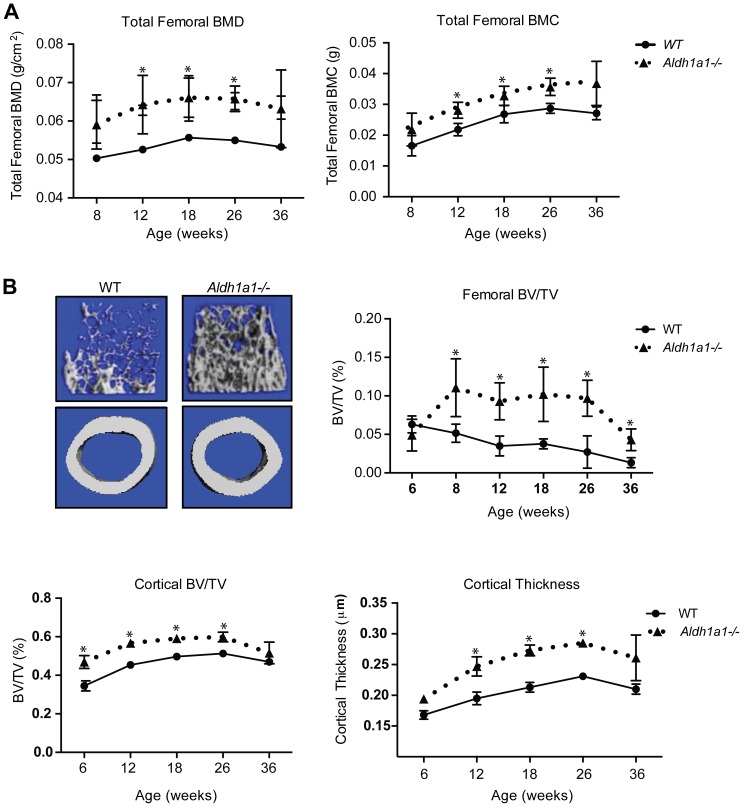
Aldh1a1 deficiency increases trabecular and cortical bone density by bone densitometry and micro CT (µCT). A. Total femoral bone mineral density (g/cm^2^) and bone mineral content (g) by DEXA (PIXImus) of age and sex-matched WT and *Aldh1a1^−/−^* female mice on standard chow diet at multiple time points including 8 weeks (WT n = 5, *Aldh1a1^−/−^* n = 5), 12 weeks (WT n = 20, *Aldh1a1^−/−^* n = 18), 18 weeks (WT n = 10, *Aldh1a1^−/−^* n = 10), 26 weeks (WT n = 10, *Aldh1a1^−/−^* n = 9), and 36 weeks (WT n = 4, *Aldh1a1^−/−^* n = 4), B. µCT of 12 week-old female WT and *Aldh1a1^−/−^* mice on standard chow diet demonstrated a significant increase in femoral trabeculations and cortical density (top left panels). µCT was performed at 6 weeks (WT n = 5, *Aldh1a1^−/−^* n = 5), 8 weeks (WT n = 5, *Aldh1a1^−/−^* n = 5), 12 weeks (WT n = 20, *Aldh1a1^−/−^* n = 18), 18 weeks (WT n = 10, *Aldh1a1^−/−^* n = 10), 26 weeks (WT n = 10, *Aldh1a1^−/−^* n = 9), and 36 weeks (WT n = 4, *Aldh1a1^−/−^* n = 4). These studies demonstrated these differences arise as early as 6–8 weeks of age and persist through 36 weeks of age. *p<0.05.

To test the hypothesis that Aldh1a1 functions as a determinant of bone mineral density, we performed skeletal phenotyping of age and sex-matched WT C57Bl6 and *Aldh1a1^−/−^* mice at multiple time points (8, 12, 18, 26, 36 weeks). Dual x-ray absorptive densitometry (DEXA) demonstrated that *Aldh1a1^−/−^* female mice had higher total femoral bone mineral density (BMD) and bone mineral content (BMC) than age-matched WT controls (Figure 2A). As early as 12 weeks of age, *Aldh1a1^−/−^* mice had statistically significant increases in total femoral BMD (0.0643±0.0076 vs. 0.0526±0.0013, p = 8.82×10^−6^) and BMC (0.281±0.0026 vs. 0.218±0.0020, p = 1.75×10^−9^). These patterns persisted through 36 weeks of age. *Aldh1a1^−/−^* mice exhibited a similar pattern on micro computed tomography (µCT) (Figure 2A). Compared to WT controls, *Aldh1a1^−/−^* mice had increased trabecular and cortical bone mass as well as micro-architectural changes suggestive of increased osteoblastic activity *in vivo* (Figure 2B and Table S1 and S2). By 12 weeks of age, *Aldh1a1^−/−^* mice demonstrated significant increases in femoral trabecular bone volume/total volume (BV/TV; 0.0929±0.0240 vs. 0.0349±0.0128, p = 1.42×10^−9^), femoral cortical BV/TV (0.567±0.023 vs. 0.454±0.0171, p = 5.23×10^−8^), and cortical thickness (0.247±0.0157 vs. 0.195±0.0104 µM, p = 3.76×10^−6^). These findings were accompanied by increases in trabecular number (TbN; 4.661±0.604 vs. 3.217±0.158, p = 0.0046) and a lower structure model index (SMI; 2.745±0.203 vs. 3.548±0.321, p = 0.035) as early as 8 weeks of age.

**Figure 3:**
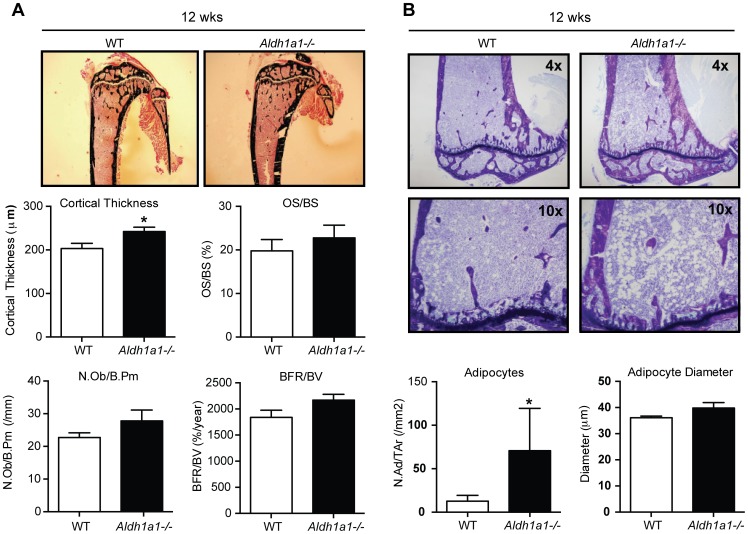
Histomorphometry of *Aldh1a1^−/−^*mice demonstrates increased cortical bone density. A. Static histomorphometry demonstrated significant increases in cortical thickness in 12 week old *Aldh1a1^−/−^* mice (n = 5) compared to WT controls (n = 5). There were no significant differences in trabecular microarchitecture and bone density; however, a trend toward higher osteoid surface to total surface area (OS/OV) and bone formation rate per bone volume (BFR/BV) on dynamic histomorphometry was noted. B. Marrow adipocyte content of *Aldh1a1^−/−^*mice. Toluidine blue staining (top panels) and adipocyte ghost quantification demonstrated higher marrow adipocyte content in *Aldh1a1^−/−^* mice compared to WT controls. The average adipocyte diameter was not significantly different between WT and *Aldh1a1^−/−^*mice. *p<0.05.

To evaluate bone remodeling, static and dynamic histomorphometric analysis was performed on 12 week-old female WT and *Aldh1a1^−/−^* mice (Figure 3 and Table 1). Consistent with the non-invasive phenotyping studies above, *Aldh1a1^−/−^* mice manifest greater cortical thickness (242±10 vs 203±12 µm, p = 0.041), as well as a trend toward higher osteoid surface per bone surface (22.77±2.91 vs. 19.80±2.58%, p = 0.451) (Figure 2A). In addition, *Aldh1a1^−/−^* mice exhibited a trend toward higher osteoblast numbers as seen on number of osteoblasts per bone perimeter (NOb/B.Pm; 27.79±3.32 vs. 22.72±1.43/mm, p = 0.199) and bone formation rate (2169±112 vs. 1838±138%/year, p = 0.0997) (Figure 3A). Interestingly, histological sections from *Aldh1a1^−/−^* mice revealed significantly higher total adipocyte numbers (NAd/TAr) versus WT controls (70.74±48.72 vs. 12.64±6.66, p = 0.0296) while adipocyte diameter was unchanged (Figure 3B). Given these findings, we then examined serum markers of osteoblast function and bone turnover in age and sex-matched WT and *Aldh1a1^−/−^* mice (Table 2). Among the markers assayed, *Aldh1a1^−/−^* mice demonstrated the most consistent differences in serum insulin-like growth factor one (IGF-1), which has previously been reported as an important determinant of bone development and growth [35]–[37]. *Aldh1a1^−/−^* mice had statistically significant increases in serum IGF-1 levels at 12 and 36 weeks and clear trends toward higher serum IGF-1 levels at 18 and 26 weeks of age. Serum osteocalcin (OCN) was not significantly different at multiple developmental time points. Serum Trap5B was higher only in 26 week-old *Aldh1a1^−/−^* mice compared to WT controls, but was not elevated at 12, 18, or 36 weeks of age. We also measured serum C-terminal telopeptides of type 1 collagen, a marker of bone resorption, (RatLaps^Tm^, KeraFast) in 18 week-old WT and *Aldh1a1^−/−^* mice, and found no significant differences (data not shown).

**Table 1:** Static and Dynamic Histomorphometeric analysis of 12 week-old WT control and *Aldh1a1^−/−^* mice.

Parameters	WT (n = 5)	*Aldh1a1^−/−^* (n = 5)	*p* value
BV/TV (%)	5.61±0.66	4.09±1.00	0.239
Tb.Th (µm)	32.84±1.67	28.03±1.82	0.087
Tb.N (/mm)	1.70±0.13	1.41±0.24	0.330
MS/BS (%)	37.72±2.60	33.92±1.86	0.269
Tb.Sp (µm)	571±46	748±99	0.143
MAR (µm/day)	2.21±0.18	2.47±0.19	0.340
BFR/BS (µm^3^/µm^2^/year)	303±27	307±35	0.918
BFR/BV (%/year)	104±15	93±29	0.748
BFR/TV (%/year)	1838±138	2169±112	0.0997
Ob.S/BS (%)	28.10±2.95	29.50±1.79	0.695
N.Ob/T.Ar (/mm^2^)	66.43±4.79	65.79±7.49	0.944
N.Ob/B.Pm (/mm)	22.72±1.43	27.79±3.32	0.199
OV/TV (%)	0.204±0.021	0.197±0.029	0.850
OS/BS (%)	19.80±2.58	22.77±2.91	0.466
O.Th (µm)	3.80±0.17	3.85±0.32	0.888
Oc.S/BS (%)	2.37±0.39	1.75±0.55	0.389
N.Oc/T.Ar (/mm^2^)	2.63±0.37	1.79±0.64	0.290
N.Oc/B.Pm (/mm)	0.89±0.11	0.66±0.22	0.385
ES/BS (%)	0.98±0.31	0.38±0.25	0.172
Cortical thickness (µm)	203±12	242±10	0.041

**Table 2:** Serum Markers.

Serum Marker	12 weeks	18 weeks	26 weeks	36 weeks
IGF-1 (ng/mL)	WT: 470.2±102.8 (n = 22)	WT: 488.3±121.0 (n = 16)	WT: 568.7±68.8 (n = 4)	WT: 486.8±85.5 (n = 4)
	*Aldh1a1^−/−^*: 577.5±180.7* (n = 20)	*Aldh1a1^−/−^* : 597.5±206.6 (n = 15)	*Aldh1a1^−/−^* : 649.6±247.7 (n = 9)	*Aldh1a1^−/−^*: 779.0±139.1* (n = 3)
OCN (ng/mL)	WT: 63.4±56.8 (n = 16)	WT: 41.3±29.1 (n = 16)	WT: 35.1±22.8 (n = 4)	WT: 16.1±7.0 (n = 4)
	*Aldh1a1^−/−^*: 46.5±31.7 (n = 15)	*Aldh1a1^−/−^* : 44.1±22.9 (n = 16)	*Aldh1a1^−/−^* : 20.4±14.4 (n = 9)	*Aldh1a1^−/−^*: 8.2±7.5 (n = 3)
Trap5B (U/L)	WT: 7.4±1.0 (n = 16)	WT: 7.3±2.4 (n = 11)	WT: 5.1±0.5 (n = 5)	WT: 6.5±1.2 (n = 4)
	*Aldh1a1^−/−^* : 7.9±2.5 (n = 16)	*Aldh1a1^−/−^* : 11.1±3.6 (n = 11)	*Aldh1a1^−/−^* : 7.3±1.6* (n = 9)	*Aldh1a1^−/−^*: 10.8±5.6 (n = 3)

* p<0.05.

### Aldh1a1 Deficiency Increases MSC Osteoblastogenesis and Adipogenesis *in vitro*

**Figure 4:**
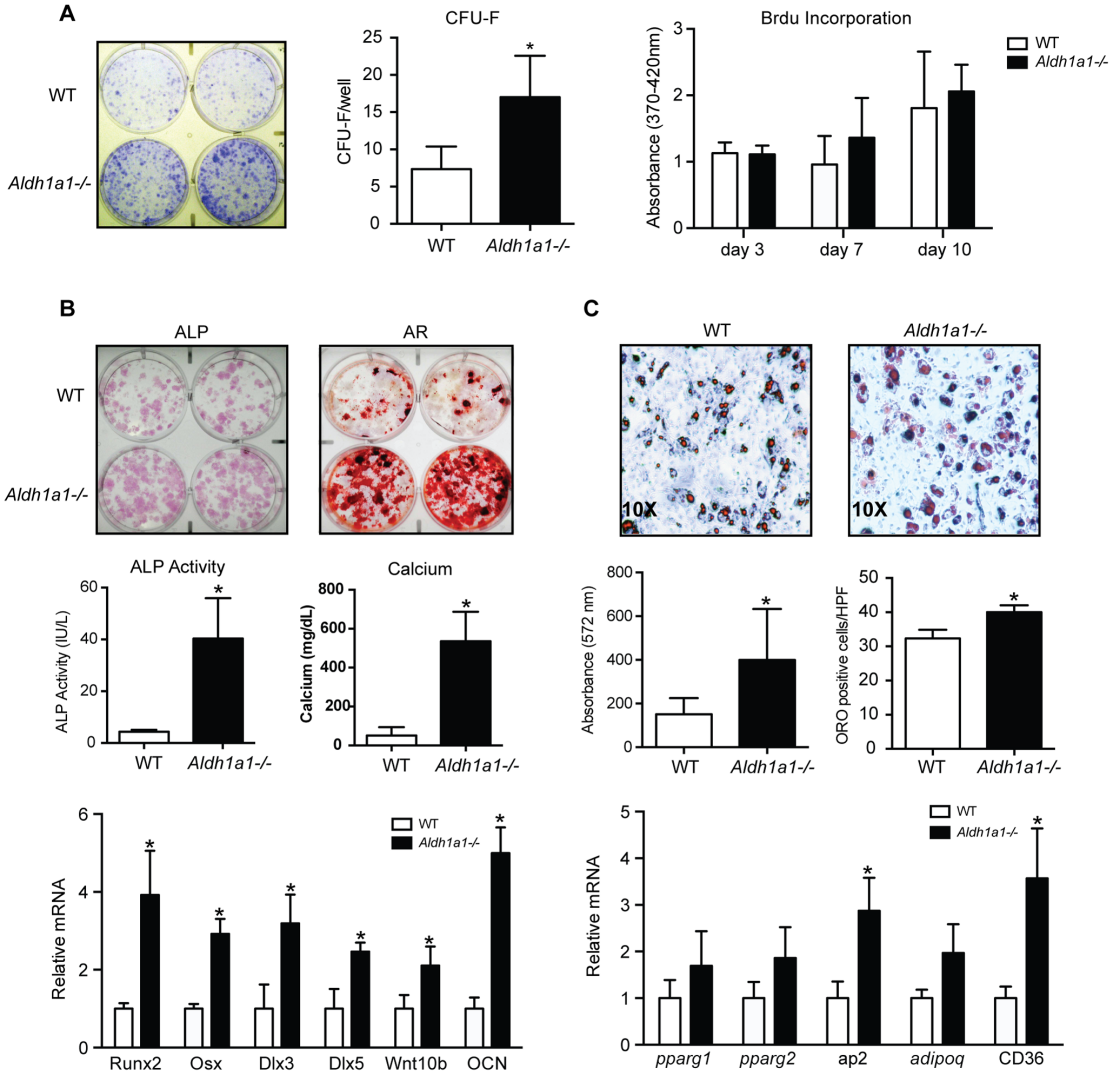
Primary *Aldh1a1^−/−^* MSCs demonstrate enhanced osteoblastogenesis and adipogenesis *in vitro*. A. Colony forming unit-fibroblast (CFU-F) assays. *Aldh1a1^−/−^* marrow stromal cultures form more CFU-F by crystal violet staining and enumeration of Giemsa stained colonies after 7 days. Brdu incorporation assays showed no significant differences in proliferation between WT and *Aldh1a1^−/−^* cultures. B. *In vitro* MSC osteoblastogenesis differentiation assays. Primary *Aldh1a1^−/−^* marrow stromal cultures treated with ascorbic acid (25 µg/mL) and beta-glycerol phosphate (0.1 M) for 7 days expressed more alkaline phosphatase (ALP) as measured by histological staining and ALP activity assays compared to WT cultures. At 14 days, *Aldh1a1^−/−^* osteogenic cultures also demonstrated greater mineralization by alizarin red (AR) staining and calcium measurements. Gene expression analysis after 7 days of osteoblast differentiation showed higher expression of Runx2, Osx, Dlx 3, Dlx 5, Wnt 10b, and OCN in *Aldh1a1^−/−^* cultures. C. *In vitro* MSC adipogenesis differentiation assays. Adherent primary *Aldh1a1^−/−^* marrow stromal cells induced to undergo adipogenesis formed more oil red O (ORO) positive cells and accumulated more intracellular lipid than WT controls. Gene transcript analysis showed corresponding increases in adipogenic markers such as aP2 and CD36. For all the cellular assays, each experiment was performed with experimental triplicates and repeated a minimum of 3 times. The data presented are from 1 representative experiment. *p<0.05.

Given the results of the phenotyping studies and histomorphometric analysis, we sought to determine whether Aldh1a1 deficiency modulated fundamental aspects of bone marrow MSC function and differentiation. To do this, we performed *in vitro* CFU-F assays, which provide a measure of MSCs within the bone marrow niche. Primary marrow stromal cultures from *Aldh1a1^−/−^* mice formed more CFU-F as compared to WT as quantified by crystal violet staining and CFU-F enumeration by Giemsa staining and microscopy (Figure 4A). To exclude that this increase in CFU-F was due to differences in proliferation, BrdU incorporation studies were undertaken, and these assays revealed no difference in proliferation between WT and *Aldh1a1^−/−^* cultures (Figure 4A).

We then examined the effect of Aldh1a1 deficiency on MSC differentiation by conducting *in vitro* MSC osteoblastogenesis and adipogenesis assays using primary marrow stromal cultures (Figure 4B, C). *Aldh1a1^−/−^* primary marrow stromal cultures treated with ascorbic acid (50 µg/mL) and beta-glycerol phosphate (10 mM) demonstrated enhanced MSC osteoblastogenesis as measured by alkaline phosphatase staining and activity (40.34±15.57 vs. 4.38±0.73 IU/L, p = 0.016) after 7 days of differentiation (Figure 4B). In addition, *Aldh1a1^−/−^* osteoblastogenesis cultures displayed greater mineralization by alizarin red staining and calcium content (536.0±151.20 vs. 50.94±42.81 mg/dL/mg of protein, p = 0.0059) after 14 days of differentiation. Consistent with these findings, *Aldh1a1^−/−^* osteoblastogenesis cultures expressed higher levels of key osteogenic transcription factors and signaling proteins, including Runx2, Osx, wingless-type MMTV integration site family member 10b (Wnt10b), as well as OCN, a marker of mature osteoblasts.

Given the observed increase in bone marrow adiposity *in vivo* in *Aldh1a1^−/−^* mice, we next studied *in vitro* MSC adipogenesis as a function of Aldh1a1. Primary *Aldh1a1^−/−^* marrow stromal cultures treated with a standard adipogenic cocktail (insulin, dexamethasone, IBMX, rosiglitazone) formed more adipocytes by ORO staining. In addition, *Aldh1a1^−/−^* marrow adipogenesis cultures accumulated more intracellular neutral lipids than WT controls by quantification of AdipoRed staining using absorbance measurements (330.17±66.2 vs. 141.08±21.12, p = 0.0080) (Figure 4C). Mature adipocyte phenotypic markers such as fatty acid binding protein 4 (aP2) and CD36 expression were also induced significantly more in the primary *Aldh1a1^−/−^* adipogenesis cultures.

### Aldh1a1 Deficiency Induces BMP2 in Primary MSCs and in the Skeleton *in vivo*

**Figure 5:**
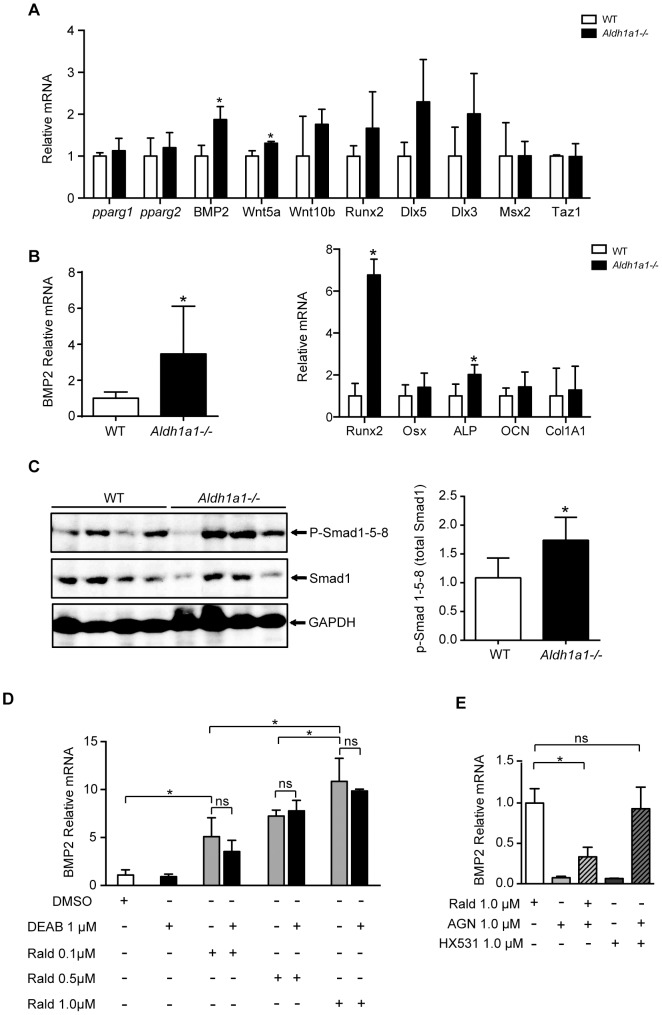
Aldh1a1 deficiency induces BMP2 expression in bone cells *in vitro* and *in vivo*. A. Gene expression analysis of primary *Aldh1a1^−/−^* marrow stromal cultures. *Aldh1a1^−/−^* marrow stromal cultures express higher levels of BMP2 *in vitro*. B. BMP2 expression in long bones (femur and tibia). BMP2 mRNA was expressed at higher levels in the long bones of *Aldh1a1^−/−^* mice (n = 10) compared to WT (n = 8). In addition, downstream transcriptional targets of BMP2 activated Smad proteins such as Runx2 and ALP are also induced in bones from *Aldh1a1^−/−^* mice compared to WT controls. C. Smad phosphorylation in long bones of *Aldh1a1^−/−^* mice. Smad 1,5,8 proteins were phosphorylated to a greater extent in the long bones of *Aldh1a1^−/−^* mice (n = 4) compared to WT (n = 4) by western blot and densitometry analysis. Phosphorylated Smad protein levels were normalized to total Smad 1 levels. D. Rald effects on BMP2 expression in primary marrow stromal cultures. Rald induced BMP2 expression in WT primary marrow stromal cultures after 24 hours of treatment at concentrations of 100 nM, 500 nM, and 1 µM. Rald-mediated induction of BMP2 in WT marrow stromal cultures was not blocked by the Aldh1 inhibitor DEAB (1 µM). E. The RAR antagonist AGN (1 µM), but not the RXR antagonist HX531 (1 µM), significantly attenuated Rald-mediated induction of BMP2. The data presented are from one representative experiment. The studies were performed with experimental triplicates and validated in 3 independent experiments. *p<0.05.

To investigate molecular mechanisms involved in the concomitant increase in both osteoblastogenic and adipogenic *Aldh1a1^−/−^* MSCs, we examined mRNA expression patterns of key proteins and transcription factors involved in MSC lineage determination (Figure 5A). Compared to WT controls, undifferentiated primary *Aldh1a1^−/−^* marrow stromal cultures selectively expressed significantly higher levels of BMP2 (p = 0.021) in the marrow microenvironment. BMP2 plays an essential role in bone formation primarily through Smad and p-38/MAP signaling pathways [21], [38]. BMP2 can also promote MSC adipogenesis *in vitro* through Smad-dependent mechanisms [23], [38]. This approximately two-fold increase in BMP2 mRNA expression in *Aldh1a1^−/−^* marrow stromal cultures was accompanied by higher expression of several downstream targets of BMP2, including Runx2, Dlx3, and Dlx5. The expression of other BMPs implicated in MSC differentiation, such as BMP4 and 7 as well as BMP receptors (BMPRIA, BMPR1B, BMPRII), was not significantly different between WT and *Aldh1a1^−/−^* MSCs (data not shown). Other transcription factors that modulate MSC adipogenesis specifically, such as PPARγ1, PPARγ2 (Figure 5A) and C/EBPs (data not shown), were not differentially expressed in *Aldh1a1^−/−^* marrow stromal cultures.

To determine whether similar gene expression patterns were present in the skeleton of *Aldh1a1^−/−^* mice *in vivo*, mRNA transcript analysis was performed on RNA isolated from whole bone (femurs and tibias) from matched, chow-fed WT and *Aldh1a1^−/−^* mice. BMP2 expression was approximately three fold higher in the long bones of the *Aldh1a1^−/−^* mice (p = 0.0440) versus WT controls (Figure 5B). Osteogenic downstream targets of BMP2-Smad pathways, including Runx2 and ALP, were also expressed at higher levels in the long bones of *Aldh1a1^−/−^* mice. BMP2 signaling is known to promote Smad phosphorylation [38]. To assess whether the increased BMP2 mRNA expression seen here was accompanied by functional distal signaling changes, western blotting was performed to determine Smad phosphorylation status (Figure 5C). Phosphorylated Smad 1–5–8 proteins to total Smad 1 protein ratios were significantly increased in femurs and tibias of *Aldh1a1^−/−^* mice as seen on densitometry (1.73±0.34 vs. 1.09±0.40; p = 0.048). Since Aldh1a1 controls the levels of key retinoid metabolites that modulate retinoid receptor activity, we also examined skeletal expression of a cassette of retinoid-regulated genes. Cyclin-dependent kinase inhibitor 1a (Cdkn1), a canonical retinoid-regulated target gene [39], [40] with a retinoic acid response element in its promoter [41], is expressed at higher levels in the femurs and tibias of *Aldh1a1^−/−^* mice compared to controls (Figure S1A). In addition, the retinoid-regulated genes transglutaminase 2 (Tgm2) [42] and osteopontin (OPN) [43] were also significantly induced in the long bones of *Aldh1a1^−/−^* mice. Other retinoid target genes, including uncoupling protein 1 (UCP-1) [44] and retinoic acid receptor beta (RARβ) [43] trended toward increased levels as well (Figure S1A). In aggregate, these data suggest that Aldh1a1 deficiency enforced a fundamental shift in retinoid signaling in bone *in vivo*.

Given that Aldh1a1 converts Rald to ATRA specifically, we next tested the effects of Rald stimulation on WT MSCs to further examine potential mechanisms involved in induction of BMP2 expression by Aldh1a1 deficiency. Primary WT marrow stromal cultures stimulated with Rald at concentrations of 100 nM, 500 nM, and 1 µM for 24 hours (Figure 5D) expressed significantly higher BMP2 mRNA levels compared to DMSO-treated cultures (statistically significant based on a two way ANOVA test and post hoc analysis with Tukey multiple comparison test with alpha<0.05). ATRA increased BMP2 expression to a similar extent as Rald at doses of 500 nM and 1 µM. At the lower concentration of 100 nM, ATRA was more potent than Rald (Figure S1B). To determine whether Rald exerted its effects on BMP2 expression independent of its conversion to ATRA, we then co-treated WT marrow stromal cultures with Rald (100 nM, 500 nM, and 1 µM) and the Aldh1 inhibitor diethylaminobenzaldehyde (DEAB) [44], which blocks the generation of ATRA from Rald (Figure 5D). Interestingly, Rald retained its transcriptional effects on BMP2 expression even in the presence of the Aldh1 inhibitor DEAB (1 µM). To further investigate whether Rald effects on BMP2 were dependent on RAR or the retinoid x receptor (RXR), Rald stimulations were repeated in the presence of the pan RAR-antagonist AGN 193109 (AGN) [45] or the RXR antagonist HX531 [46]. The pan RAR-antagonist AGN significantly attenuated Rald induction of BMP2 while the RXR antagonist HX531 had no effect (Figure 5E). In aggregate, these data suggest that Rald stimulates BMP2 expression in MSCs in a RAR-dependent manner, independent of ATRA formation.

## Discussion

Retinoids exert complex, important post-natal skeletal effects. Recent data has shown that Rald has distinct transcriptional effects independent of its conversion to ATRA [27]. Thus, the enzymatic machinery controling levels of Rald, ATRA, and other retinoid metabolites, is poised to regulate fundamental aspects of osteogenic programs. Here we demonstrate that Aldh1a1, the primary enzyme responsible for converting Rald to ATRA in adult animals, plays an important role in bone metabolism. Non-invasive imaging with PIXImus and µCT revealed that *Aldh1a1^−/−^* mice have higher femoral bone mineral density at multiple developmental time points. Although µCT identified significant trabecular and cortical microarchitectural changes in *Aldh1a1^−/−^* mice (Figure 1), histomorphometry uncovered predominant increases in cortical thickness only (Figure 2). Thus, Aldh1a1 may play a unique role in cortical bone as a result of local factors determining enzymatic activity, retinoid levels, or the action of retinoid-modulated nuclear receptors.

Interestingly, the consistent increase in cortical bone mass observed in *Aldh1a1^−/−^* mice across imaging modalities was coupled to higher bone marrow adiposity. These data suggest that Aldh1a1 deficiency alters differentiation programs in MSCs, the common progenitor of osteoblasts and adipocytes in the marrow. Primary *Aldh1a1^−/−^* MSCs in marrow stromal cultures manifest higher BMP2 mRNA expression and enhanced osteoblastogenic and adipogenic differentiation as compared to WT primary MSCs (Figure 4). BMP2 was originally described as a potent osteo-inductive factor [20], [21]. Unlike other signaling proteins and transcription factors such as Taz, PPARγ, and Wnts that modulate MSC lineage determination in a reciprocal fashion, BMP2 may stimulate both osteoblastogenesis and adipogenesis in MSCs [38]. As such, BMP2 is an appealing candidate for coordinating both MSC osteoblastogenesis and adipogenesis responses seen *in vitro* and *in vivo* in Aldh1a1 deficiency.

BMP2 signals through Smad transcription factors that are phosphorylated and activated by the BMP receptor II. Long bones of *Aldh1a1^−/−^* mice had higher expression of BMP2 and its downstream targets, such as Runx2 and ALP, as well as increased Smad 1–5–8 phosphorylation *in vivo* (Figure 5). Although Aldh1a1 has various reported functions, its primary biologic role is conversion of Rald to ATRA, as supported by the higher Rald levels found in *Aldh1a1^−/−^* mice [28], [34]. Rald potently induced BMP2 mRNA expression in primary WT marrow stromal cultures (Figure 5), consistent with the possibility that Rald drives the changes in bone in Aldh1a1 deficiency. Based on the data presented here, Rald appears to induce BMP2 through direct RAR-dependent transcriptional actions (Figure 5F). A well-established RAR antagonist (AGN) completely blocked Rald-mediated induction of BMP2 expression in MSCs while an RXR antagonist (HX531) had no such effect, suggesting nuclear receptor selectivity. Previous work indicates that RAR may modulate key transcriptional events at the BMP2 promoter in F9 embryonal cells [47]. In this cellular context, RAR forms a co-regulatory complex with SP1 at the BMP2 promoter that represses transcription. Upon ATRA stimulation, the RAR-SP1 complex dissociates from the promoter, thereby de-repressing BMP2 expression. Although these prior findings and our present data suggest that Rald accumulation in Aldh1a1 deficiency may modulate BMP2 promoter activity in MSCs through similar mechanisms, the bone phenotype in Aldh1a1 deficiency raises the possibility of other levels of potential control through Rald and/or Aldh1a1– a topic of considerable interest for future studies.

In examining factors responsible for increased cortical bone density in *Aldh1a1^−/−^* mice, we considered differences in lean mass, which correlates with cortical thickness [48]–[51]. However, chow-fed matched WT and *Aldh1a1^−/−^* female mice have similar total body weights, fat mass, and lean mass [52]. Another potential contributor to the cortical bone phenotype of the *Aldh1a1^−/−^* mice is serum IGF-1, which has also been implicated in selective increases in cortical thickness through modulation of periosteal apposition during growth [53]. Indeed, *Aldh1a1^−/−^* mice manifest higher serum IGF-1 levels at 12 and 36 weeks of age (Table 2), suggesting that the cortical bone phenotype of *Aldh1a1^−/−^*mice may, at least in part, be the result of higher levels of IGF-1.

Aldh1a1 deficiency may also shed light on age-related bone loss. Cortical thinning is a key feature of human Type II osteoporosis [54], [55], as well as age-related bone loss in murine models [56]. Female C57Bl/6 mice lose significant cortical bone as early as 7 months of age [57]. *Aldh1a1^−/−^*mice, which have significantly higher cortical bone thickness and cortical BV/TV by µCT as early as 6 weeks of age, appear to resist age-related bone loss through at least 36 weeks of age (Figure 1). Aldh1a1 deficiency may promote this phenotype through multiple mechanisms. Higher serum IGF-1 levels in *Aldh1a1^−/−^* mice may confer protection against age-related cortical bone loss through selective effects on cortical periosteal apposition growth. In addition, osteoblast insufficiency has been strongly implicated as a major factor in age-related bone loss [58]. Our data indicate that Aldh1a1 deficiency drives enhanced osteoblast activity and mineralization. Finally, given that bone resorption is increased in osteoporosis [59], the higher cortical thickness in *Aldh1a1^−/−^* mice could result from osteoclast effects. Although similar osteoclast numbers and serum markers of bone turnover such as Trap5B and RatLaps were observed in matched WT and *Aldh1a1^−/−^* mice, a trend toward a lower eroded surface/bone surface (0.98±0.31% vs. 0.38±0.25%, p = 0.17; Table 2) raises the possibility that a functional osteoclast defect may contribute to the cortical bone phenotype in Aldh1a1 deficiency.

Of note, a recent study reported that deficiency of acetaldehyde dehydrogenase, which oxidizes acetaldehyde during ethanol metabolism, results in greater cortical bone thickness, with increased expression of osteogenic genes including BMP2, Runx2, Osx, and Wnt 5a; changes in bone marrow adiposity were not noted [60]. Despite the similarity in the skeletal phenotypes in acetaldehyde dehydrogenase and Aldh1a1 deficiency, limited overlap exists between the substrates or biological roles of Aldh1a1 and acetaldehyde dehydrogenase. Acetaldehyde dehydrogenase oxidizes acetaldhehyde but does not bind or metabolize Rald [61], [62]. Although Aldh1a1 can bind acetaldehyde, it does so with a 50-fold lower affinity than Rald, its primary substrate [63]. Nevertheless, the shared cortical bone phenotypes seen in these models raise intriguing questions regarding convergent distal pathways worthy of further study while supporting the notion that key metabolic enzymes and their substrates may play important, overlooked roles in determining bone formation.

Consistent with this concept, we provide here *in vitro* and *in vivo* evidence that Aldh1a1 deficiency increases cortical bone density. These changes appear to result from increased BMP2 expression and shifts in MSC lineage fate decisions that drive coordinated changes in bone density and marrow adiposity in Aldh1a1 deficient mice. These results suggest further consideration is warranted regarding the regulation of Aldh1a1 and Rald in metabolic disorders like osteoporosis, diabetes, and obesity that are characterized by changes in both bone density and marrow adiposity.

## Supporting Information

Figure S1 A. Gene expression patterns of retinoid targets in femurs and tibias of matched WT and *Aldh1a1^−/−^* mice. *Aldh1a1^−/−^* mice express higher levels of the retinoid target cdkn1a in their femurs and tibias compared to WT. B. Effects of ATRA on BMP2 expression in primary WT marrow stromal cultures. ATRA induced BMP2 expression in WT primary marrow stromal cultures after 24 hours of treatment at concentrations of 100 nM, 500 nM, and 1 µM. * p<0.05.
(TIF)

Table S1 (DOCX)

Table S2 (DOCX)
