# Supplementary figures and images for: Deficiency of Retinaldehyde Dehydrogenase 1 Induces BMP2 and Increases Bone Mass In Vivo
Source: PLoS One. 2013 Aug 9;8(8):e71307. doi: 10.1371/journal.pone.0071307 (PMC3739807; doi:10.1371/journal.pone.0071307)

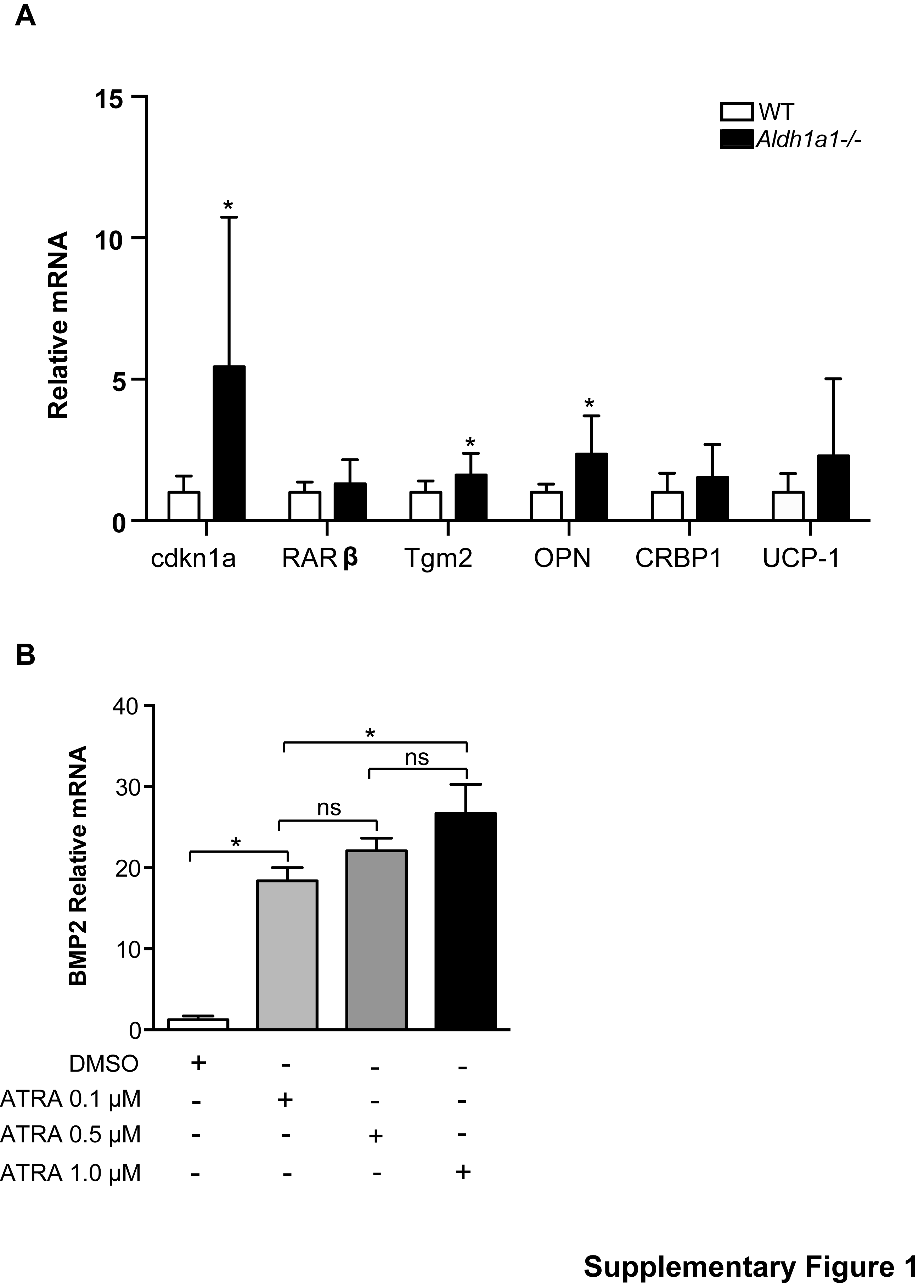

Supplement: Figure S1 — A. Gene expression patterns of retinoid targets in femurs and tibias of matched WT and Aldh1a1−/− mice. Aldh1a1−/− mice express higher levels of the retinoid target cdkn1a in their femurs and tibias compared to WT. B. Effects of ATRA on BMP2 expression in primary WT marrow stromal cultures. ATRA induced BMP2 expression in WT primary marrow stromal cultures after 24 hours of treatment at concentrations of 100 nM, 500 nM, and 1 µM. * p<0.05. (TIF) [file pone.0071307.s001.tif]
