# Supplementary material for: Deficiency of Retinaldehyde Dehydrogenase 1 Induces BMP2 and Increases Bone Mass In Vivo
Source: PLoS One. 2013 Aug 9;8(8):e71307. doi: 10.1371/journal.pone.0071307 (PMC3739807; doi:10.1371/journal.pone.0071307)
Supplement: Table S1 — (DOCX) [file pone.0071307.s002.docx]

Table S1: Micro CT of chow-fed age-matched female WT and *Aldh1a1^-/-^* mice

| **AGE** | **Fem BV/TV** | **Fem TbN** | **Fem TbT (μm)** | **SMI** |
| --- | --- | --- | --- | --- |
| **6 weeks**: |  |  |  |  |
| WT (n=5) | 0.0629±0.0110 | 3.521±0.137 | 0.0363±0.00193 | 2.962±0.115 |
|  |  |  |  |  |
| *Aldh1a1^-/-^* (n=5) | 0.0.049±0.0205 | 3.265±0.457* | 0.0374±0.00321 | 3.299±0.311 |
|  |  |  |  |  |
| **8 weeks**: |  |  |  |  |
| WT (n=5) | 0.0515±0.0118 | 3.217±0.158 | 0.0404±0.00128 | 3.301±0.451 |
|  |  |  |  |  |
| *Aldh1a1^-/-^* (n=5) | 0.1105±0.0375* | 4.66±0.6037* | 0.0462±0.00267 | 2.656±0.329* |
|  |  |  |  |  |
| **12 weeks**: |  |  |  |  |
| WT (n=20) | 0.0349±0.0128 | 3.039±0.293 | 0.0352±0.0033 | 3.548±0.321 |
|  |  |  |  |  |
| *Aldh1a1^-/-^* (n=18) | 0.0929±0.0240* | 4.177±0.358*** | 0.0405±0.00420* | 2.745±0.203** |
|  |  |  |  |  |
| **18 weeks**: |  |  |  |  |
| WT (n=10) | 0.0377±0.00640 | 3.140±0.206 | 0.0358±0.00291 | 3.292±0.129 |
|  |  |  |  |  |
| *Aldh1a1^-/-^* (n=10) | 0.102±0.0353* | 3.865±0.304* | 0.0396±0.00410* | 2.743±0.227* |
|  |  |  |  |  |
| **26 weeks**: |  |  |  |  |
| WT (n=10) | 0.0270±0.0210 | 2.551±0.263 | 0.0401±0.00575 | 3.41±0.229 |
|  |  |  |  |  |
| *Aldh1a1^-/-^* (n=9) | 0.0969±0.0233** | 3.410±0.299* | 0.0476±0.00370* | 2.425±0.217** |
|  |  |  |  |  |
| **36 weeks**: |  |  |  |  |
| WT (n=4) | 0.0132±0.00653 | 1.958±0.0639 | 0.0424±0.00784 | 3.554±0.175 |
|  |  |  |  |  |
| *Aldh1a1^-/-^* (n=4) | 0.0430±0.0141* | 2.408±0.273* | 0.0434±0.00836 | 2.88±0.187* |
|  |  |  |  |  |

* p<0.05; **p< 1 x 10^-8^; ***p<1 x 10^-11^
