# Supplementary material for: Deficiency of Retinaldehyde Dehydrogenase 1 Induces BMP2 and Increases Bone Mass In Vivo
Source: PLoS One. 2013 Aug 9;8(8):e71307. doi: 10.1371/journal.pone.0071307 (PMC3739807; doi:10.1371/journal.pone.0071307)
Supplement: Table S2 — (DOCX) [file pone.0071307.s003.docx]

Table S2: Micro CT of chow-fed age-matched female WT and *Aldh1a1^-/-^* mice

| **Age/Genotype** | **Femoral Cortical BV/TV** | **Fem Cortical Thickness** |
| --- | --- | --- |
| **6 weeks**: |  |  |
| WT (n=5) | 0.345±0.0258 | 0.168±0.00680 |
|  |  |  |
| *Aldh1a1^-/-^* (n=5) | 0.469±0.0335* | 0.194±0.00368* |
|  |  |  |
| **12 weeks**: |  |  |
| WT (n=10) | 0.454±0.0171 | 0.195±0.0104 |
|  |  |  |
| *Aldh1a1^-/-^* (n=8) | 0.567±0.0233** | 0.247±0.0157** |
|  |  |  |
| **18 weeks**: |  |  |
| WT (n=5) | 0.497±0.0184 | 0.213±0.00789 |
|  |  |  |
| *Aldh1a1^-/-^* (n=5) | 0.591±0.0127** | 0.273±0.00871** |
|  |  |  |
| **26 weeks**: |  |  |
| WT (n=10) | 0.5130±0.0173 | 0.231±0.000917 |
|  |  |  |
| *Aldh1a1^-/-^* (n=9) | 0.599±0.0250* | 0.285±0.00184* |
|  |  |  |
| **36 weeks**: |  |  |
| WT (n=4) | 0.470±0.0160 | 0.210±0.00826 |
|  |  |  |
| *Aldh1a1^-/-^* (n=4) | 0.516±0.0562 | 0.261±0.0371 |
|  |  |  |

* p<0.05, **p< 1 x 10^-4^
